# Dental Abnormalities in Pediatric Patients Receiving Chemotherapy

**DOI:** 10.3390/jcm13102877

**Published:** 2024-05-13

**Authors:** Tatsuya Akitomo, Masashi Ogawa, Ami Kaneki, Taku Nishimura, Momoko Usuda, Mariko Kametani, Satoru Kusaka, Yuria Asao, Yuko Iwamoto, Meiko Tachikake, Chieko Mitsuhata, Ryota Nomura

**Affiliations:** 1 Department of Pediatric Dentistry, Graduate School of Biomedical and Health Sciences, Hiroshima University, Hiroshima 734-8553, Japan; caries0@hiroshima-u.ac.jp (M.O.); kaneki@hiroshima-u.ac.jp (A.K.); nishi04@hiroshima-u.ac.jp (T.N.); musuda@hiroshima-u.ac.jp (M.U.); mrysk25@hiroshima-u.ac.jp (M.K.); higechi@hiroshima-u.ac.jp (S.K.); yuriaasao@hiroshima-u.ac.jp (Y.A.); yuko-tulip@hiroshima-u.ac.jp (Y.I.); chiekom@hiroshima-u.ac.jp (C.M.); rnomura@hiroshima-u.ac.jp (R.N.); 2 Department of Pediatric Dentistry, Hiroshima University Hospital, Hiroshima 734-8551, Japan; meikosan@hiroshima-u.ac.jp

**Keywords:** dental anomaly, pediatric cancer, chemotherapy

## Abstract

**Background:** Chemotherapy is a common treatment for pediatric cancer. Although life prognosis is improving because of advances in medical science, it is important to deal with late effects such as dental abnormalities. We investigated the association between dental abnormalities and chemotherapy by age and tooth type. **Methods:** Among the 568 patients referred to the pediatric dentistry department of our hospital, we selected 32 patients (21 male and 11 female) who received chemotherapy between the ages of 0 and 6 and underwent panoramic examination after the age of 7. We recorded the age of chemotherapy commencement, diagnosis of systemic disease, and dental abnormalities such as congenital absence, microdonts, and short-rooted teeth. **Results:** Almost half of the patients had dental abnormalities such as congenital absence, microdonts, and short-rooted teeth, but there were no significant differences in the incidence of these abnormalities by age. When we analyzed the incidence of abnormal teeth by tooth type, the incidence of congenital absence was significantly higher in premolars (5.5%) and second molars (3.9%) than in incisor or canine or 1st molar (0.4%) (*p* < 0.01). The incidence of microdonts was significantly higher in premolars (3.9%) than in incisor or canine or 1st molar (0.2%) and second molars (0.0%) (*p* < 0.05). **Conclusions:** Patients who received chemotherapy had a high prevalence of dental abnormalities, and the incidence of abnormalities varied by tooth type. It is important to maintain long-term oral care for patients who have undergone chemotherapy even after the treatment is completed.

## 1. Introduction

Childhood cancer is one of the leading causes of childhood death in the Western world, with 416,500 children aged 0–14 years diagnosed with cancer globally in 2017 [1,2]. In Japan, more than 160 hospitals provide care for approximately 2500 pediatric cancer patients each year [3]. Five-year survival rates have improved considerably over the last 40 years after the implementation of more intensive treatment in developed countries [1]. There are currently four independent treatment approaches for cancer: surgical removal, immunotherapy, radiotherapy, and chemotherapy [4]. In addition, hematopoietic stem cell transplantation (HSCT) is the only curative option for patients with severe congenital neutropenia, and high-dose chemotherapy combined with total body irradiation is one of the classic pretreatment regimens for HSCT [5,6].

Chemotherapy for pediatric oncology patients often causes dental developmental anomalies that affect future dental care [7]. Dental anomalies include abnormalities in tooth count, morphology, size, and eruption times, and chemotherapy causes irreversible morphological changes in permanent teeth, including tooth agenesis, microdontia, and short-rooted teeth [8,9,10]. Although there are some case reports about dental anomalies in pediatric patients who have received chemotherapy, they have mainly focused on a small number of patients. There are few reports of studies involving dozens to hundreds of patients.

In addition, cytotoxic molecules at the time of early dental development may result in agenesis or microdontia, and enamel defect, while the anomalies of root formation characterize effects of antineoplastic agents in the later stages of formation. Considering the sequence of dental calcification and development, children between 0 and 5 years of age seem to be at the highest risk of several dental abnormalities resulting from anticancer treatments [11]. However, there are few studies that have investigated in detail the age of chemotherapy, the prevalence of each type of dental abnormality, and the tooth type that causes the dental abnormality. We investigated dental abnormalities in pediatric patients who had received chemotherapy using panoramic radiographs. The objective of this study was to analyze the association between chemotherapy and dental abnormalities, focusing on the age of chemotherapy initiation and tooth type that caused the dental abnormality.

## 2. Materials and Methods

### 2.1. Study Selection

The study was performed in line with the STrengthening the Reporting of OBservational studies in Epidemiology (STROBE) statement [12]. This study was conducted in full adherence to the Declaration of Helsinki. The study protocol was approved by the Hiroshima University Epidemiology Research Ethics Committee (approval number: E2023-0211). Informed consent was obtained via opt-out on our hospital website, and patients who opted out were excluded. We focused on 568 patients who were referred by medical institutions to the Department of Pediatric Dentistry of Hiroshima University Hospital from 2012 to 2019. Using these 568 patients, the inclusion criteria were determined as follows: patients received perioperative oral function management; received chemotherapy before the age of 6 years; confirmed there were no duplicates using patient ID to avoid analyzing the same patient multiple times; and received panoramic examination after the age of 7 years, when the presence of teeth other than third molars could be confirmed. We also confirmed name, surname, and date of birth. In addition, the patients who had not received chemotherapy or who were receiving chemotherapy at another hospital were excluded.

### 2.2. Data Extraction

All authors performed perioperative oral function management of their patients and extracted details of sex, age of chemotherapy commencement, panoramic photograph, and diagnosis of systemic disease from medical records. One of the authors investigated dental abnormalities by checking panoramic radiographs taken after the age of 7. The findings of radiographic examination were evaluated by a single examiner who is a pediatric dental specialist, based on the medical records and the radiologist’s findings. Dental abnormalities were classified into congenital absence, microdonts, and short-rooted teeth. We selected teeth with size abnormalities based on a previous study [13]. In addition, for patients whose maxillary or mandibular bone had been removed during cancer treatment, only the teeth that could be identified were included.

### 2.3. Statistical Analysis

Statistical analyses were conducted using GraphPad Prism 9 (GraphPad Software Inc., La Jolla, CA, USA). Comparisons between males and females were performed using Fisher’s extract test. Age adjustments were made between the groups. Chi-square tests with Bonferroni correction were used to compare each part of the tooth and each age group. Differences were considered statistically significant at *p* < 0.05.

## 3. Results

### 3.1. Distribution of Subjects by Age, Sex, and Systemic Disease

**Figure 1:**
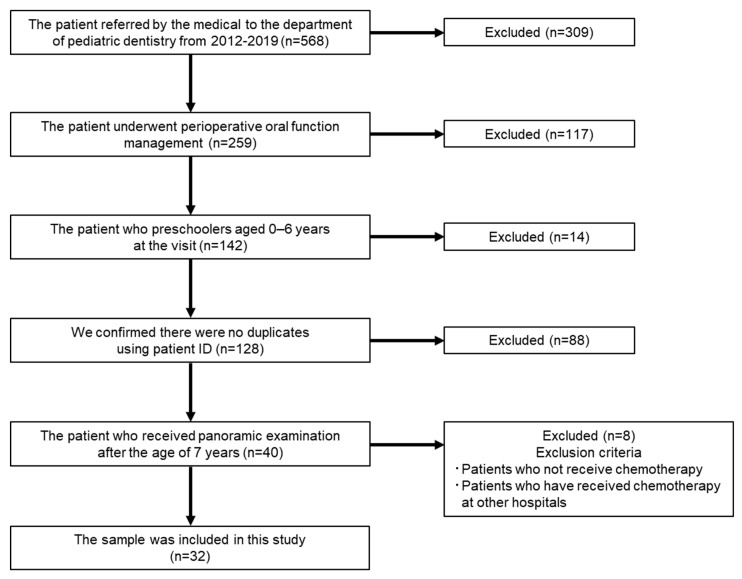
Flowchart of the study.

Figure 1 shows the flowchart for selecting subjects in this study. Of total of 568 patients, 32 people who met each inclusion criteria participated in this study. Table 1 shows the age and sex distribution of the subjects. Of the 32 subjects, 21 were male (65.6%) and 11 were female (34.4%). There were no statistically significant differences between genders regarding the timing of chemotherapy initiation. The mean age at the start of chemotherapy was 3 years and 10 months, and the median age was 4 years and 2 months. The youngest was 2 months old and the oldest was 6 years and 8 months old. Comparing the timing of chemotherapy initiation by age group, the highest rate was found in the 4-year-old group (n = 8, 25.0%), followed by the 1-year-old group (n = 6, 18.8%) and the 5-year-old group (n = 5, 15.6%).

Table 2 lists the systemic diseases of the subjects. Acute lymphoblastic leukemia was most common (n = 7, 21.9%), followed by neuroblastoma (n = 6, 18.8%). Burkitt’s lymphoma, choroid plexus carcinoma, Ewing sarcoma, medulloblastoma, and rhabdomyosarcoma were present in two patients (6.3%). Nine patients (28.1%) were classified as “Others”, which included congenital neutropenia, ependymoma, hemophagocytic syndrome, juvenile myelomonocytic leukemia, Langerhans cell histiocytosis, nephroblastoma, optic glioma, primary myelofibrosis, and retinoblastoma.

### 3.2. Prevalence of Dental Abnormalities, Number of Abnormal Teeth, and Sex Differences

The prevalence of dental abnormalities was 46.9% (n = 15), of which congenital absence was the most common at 25.0% (n = 8), followed by short-rooted teeth at 21.9% (n = 7), and microdonts at 9.4% (n = 3) (including duplicates) (Table 3). The most common number of teeth affected by chemotherapy was 1–4 (7 patients, 21.9%), followed by 5–9 and 10 or more (4 patients each: 12.5%). The average number of abnormal teeth per person was 3.63 ± 1.20. The prevalence of dental abnormalities in females was 63.6% (7 of 11 patients), higher than that of males (38.1%, 8 of 21 patients), but there was no significant difference between the two groups.

### 3.3. Prevalence of Dental Abnormalities by Age of Chemotherapy Initiation

Comparing age at the initiation of chemotherapy, the incidence of abnormal teeth was over 50% in the 0–2-year-old group and the 3–4-year-old group, which was higher than that in the 5–6-year-old group (22.2%), but there was no significant difference between the groups (Table 4). Congenital absence was most common in the 0–2-year-old group (36.4%), followed by the 3–4-year-old group (25.0%), and short-rooted teeth were most common in the 3–4-year-old group (33.3%). The number of abnormal teeth was higher in the group receiving chemotherapy at a younger age, with the average number of affected teeth per person being highest in the 0–2-year-old group (5.00 ± 2.64), followed by the 3–4-year-old group (3.67 ± 1.73) and the 5–6-year-old group (1.89 ± 1.77).

### 3.4. Prevalence of Dental Abnormalities for Each Tooth Type

When we analyzed the incidence of abnormal teeth for each tooth type, the incidence of congenital absence was significantly higher in premolar (5.5%) and 2nd molar (3.9%) than in incisors or canines or 1st molar (0.4%) (*p* < 0.01) (Table 5). The occurrence of microdonts was significantly higher in premolar (3.9%) than in incisors or canines or first molars (0.2%) and in second molars (0.0%) (*p* < 0.05). In contrast, short-rooted teeth were widely observed with no significant differences between tooth types (range 7.1% to 10.2%). There was a significant difference in the incidence of total abnormal teeth between incisor or canine or 1st molar (10.8%) and premolar (18.5%) (*p* < 0.05).

## 4. Discussion

Pediatric cancer occurs in 1 out of 10,000 people in Japan, and includes leukemia, brain tumors, and neuroblastoma. The overall survival rate of pediatric cancer has improved to approximately 70–80% as a result of advances in medical technology [14]. Because of the improving survival rate, the number of childhood cancer survivors reaching adulthood is steadily increasing. This growing population, with many years of life ahead, is subject to increased concern about the risk of late effects induced by cancer treatment at a young age, and has sparked interest in survivorship research [15].

Dental abnormalities are commonly observed as late effects of anti-neoplastic therapy in the oral cavity [16]. Therefore, dental professionals need to understand the late effects of chemotherapy and continue with long-term follow-up of the oral management of pediatric cancer patients. Although there are many studies investigating the late effects of chemotherapy, only a few have investigated the association between the age at chemotherapy initiation and dental abnormalities such as congenital absence, microdonts, or short-rooted teeth as a late effect. We conducted a follow-up survey of pediatric patients who received chemotherapy at a young age.

We selected 32 patients who were referred to our hospital from the medical department from 2012 to 2019 and who met our inclusion criteria. The third edition of the International Classification of Diseases for Oncology, which was published in 2000, classified pediatric cancer into 12 main groups, with leukemia being the most common pediatric cancer in Japan [14,17]. In the present study, acute lymphoblastic leukemia, a form of leukemia, was found to be the most common.

The crown formation of the second molar is complete at approximately 7–8 years of age [18]. Therefore, we investigated dental abnormalities in these participants using panoramic radiographs taken after the age of 7 years. Dental abnormalities were detected in 15 patients (46.9%). Bilge et al. (2018) reported that the prevalence of dental anomalies including abnormalities in tooth position or structure diagnosed by panoramic radiographs among 6–40-year-olds was 39.2% [13]. Büyükgöze-Dindar et al. (2022) studied panoramic radiographs of 43,880 admitted patients and found that the prevalence of dental anomalies was 5.2% [19]. In the present study, the prevalence of congenital absence, which was the most common abnormality, was 25.0%. According to the Japanese Society of Pediatric Dentistry, the general prevalence of congenital absence (except the third molar) was 10.1% in children, which is lower than the prevalence found among our chemotherapy patients [20]. Shum et al. (2020) investigated the prevalence of dental abnormalities in long-term survivors of childhood malignancies in New Zealand children and concluded that childhood cancer survivors had a high prevalence of developmental dental abnormalities [21]. Additionally, there are some reports of dental abnormalities in children who received chemotherapy [11,22,23,24,25]. These findings lead us to conclude that chemotherapy is a risk factor for dental abnormalities.

The frequency of congenital absence of the premolar and 2nd molar was significantly higher than that of the incisor or canine or 1st molar. In contrast, the microdonts of the premolar were significantly higher than that of the incisor or canine or 1st molar and that of 2nd molar. These differences may be associated with tooth development. Calcification of incisors, canines, or first molars has begun by the age of 1 year, while premolar calcification occurs from 1 year and 6 months to 2 years and 6 months, and second molar calcification takes place from 2 years and 6 months to 3 years [18]. Because the tooth crown and root formation progress over time, the effect on different types of teeth depends on the time of commencement of chemotherapy.

A limitation of this study was that the types of chemotherapy and administration periods were not standardized. The severity of systemic diseases may vary depending on the patient, and retreatment may be required following recurrence of the cancer. Halperson et al. (2022) reported that antineoplastic treatment that combines chemotherapy and radiotherapy appears to increase the risk of dental abnormalities; however, they did not account for outcomes that could be due to differences between patients in age, the time lapsed from diagnosis and from treatment, and the presentation of chronic health conditions [25]. Jodłowska et al. (2023) also investigated the association between dental abnormalities and their age-dependent occurrence in leukemia survivors and concluded that there was no correlation between the treatment duration of intensive therapy, the entire therapy, and the number of dental abnormalities [26]. In addition, Proc et al. (2016) reported that no statistically significant relationships between the rate or severity of dental disturbances, and the age of the patient at the beginning, the end, or during anticancer therapy, and concluded that it may result from the small sample size [24]. More extensive studies should be planned that include pediatric cancer survivors from multiple centers and focus on each age of chemotherapy commencement, treatment duration of intensive therapy, treatment modalities, or the cumulative dose of the analyzed drugs in the future.

## 5. Conclusions

It is important for dental professionals to understand these potential abnormalities and provide information to patients and medical professionals, since patients treated with chemotherapy frequently have dental abnormalities. There was no significant association between chemotherapy start time and the prevalence of abnormal teeth. We found that congenital absence after chemotherapy occurs with high frequency in premolar and 2nd molar, and microdonts occur with high frequency in premolar. Long-term follow-up is necessary because patients with dental abnormalities sometimes need prosthodontic or conservative treatment [27]. Furthermore, continuous dental support after the treatment is completed leads to better general oral health, which improves patients’ quality of life [28].

## Figures and Tables

**Table 1:** Distribution of subjects by age and gender.

Age	Male (n = 21)	Female (n = 11)	Total (n = 32)
0-year-old	0 (0.0%)	2 (18.2%)	2 (6.3%)
1-year-old	2 (9.5%)	4 (36.4%)	6 (18.8%)
2-years-old	2 (9.5%)	1 (9.1%)	3 (9.4%)
3-years-old	3 (14.3%)	1 (9.1%)	4 (12.5%)
4-years-old	6 (28.6%)	2 (18.2%)	8 (25.0%)
5-years-old	5 (23.8%)	0 (0.0%)	5 (15.6%)
6-years-old	3 (14.3%)	1 (9.1%)	4 (12.5%)

**Table 2:** Systemic diseases of samples.

Diagnosis	Total
Acute lymphoblastic leukemia	7 (21.9%)
Neuroblastoma	6 (18.8%)
Burkitt’s lymphoma	2 (6.3%)
Choroid plexus carcinoma	2 (6.3%)
Ewing sarcoma	2 (6.3%)
Medulloblastoma	2 (6.3%)
Rhabdomyosarcoma	2 (6.3%)
Others	9 (28.1%)
Total	32 (100.0%)

**Table 3:** Prevalence of dental abnormalities by gender.

	The Type or the Number of Abnormal Teeth	Male (n = 21)	Female (n = 11)	Total (n = 32)
The type of dental abnormalities (including duplicates)	Congenital absence	4 (19.0%)	4 (36.4%)	8 (25.0%)
	Microdonts	1 (4.8%)	2 (18.2%)	3 (9.4%)
	Short-rooted teeth	5 (23.8%)	2 (18.2%)	7 (21.9%)
	Total ^a^	8 (38.1%)	7 (63.6%)	15 (46.9%)
The number of abnormal teeth	1–4	3 (14.3%)	4 (36.4%)	7 (21.9%)
	5–9	2 (9.5%)	2 (18.2%)	4 (12.5%)
	10≤	3 (14.3%)	1 (9.1%)	4 (12.5%)
	Total	8 (38.1%)	7 (63.6%)	15 (46.9%)
	Average ^b^	3.52 ± 2.47	3.82 ± 1.83	3.63 ± 1.20

^a^ One subject can have multiple dental abnormalities, and the total does not match the sum of congenital absence, microdonts, and short-rooted teeth. ^b^ Average was expressed as the mean ± standard error.

**Table 4:** Prevalence of dental abnormalities by age of chemotherapy start time.

	The Type or the Number of Abnormal Teeth	0–2 Years (n = 11)	3–4 Years (n = 12)	5–6 Years (n = 9)	Total (n = 32)
The type of dental abnormalities (including duplicates)	Congenital absence	4 (36.4%)	3 (25.0%)	1 (11.1%)	8 (25.0%)
	Microdonts	2 (18.2%)	1 (8.3%)	0 (0.0%)	3 (9.4%)
	Short-rooted teeth	2 (18.2%)	4 (33.3%)	1 (11.1%)	7 (21.9%)
	Total ^a^	6 (54.5%)	7 (58.3%)	2 (22.2%)	15 (46.9%)
The number of abnormal teeth	1–4	3 (27.3%)	3 (25.0%)	1 (11.1%)	7 (21.9%)
	5–9	1 (9.1%)	3 (25.0%)	0 (0.0%)	4 (12.5%)
	10≤	2 (18.2%)	1 (8.3%)	1 (11.1%)	4 (12.5%)
	Total	6 (54.5%)	7 (58.3%)	2 (22.2%)	15 (46.9%)
	Average ^b^	5.00 ± 2.64	3.67 ± 1.73	1.89 ± 1.77	3.63 ± 1.20

^a^ One subject can have multiple dental abnormalities, and the total does not match the sum of congenital absence, microdonts, and short-rooted teeth. ^b^ Average was expressed as the mean ± standard error.

**Table 5:** Prevalence of dental abnormalities by tooth type.

	Tooth Type		
The Type of Abnormal Teeth	Incisor or Canine or 1st Molar (n = 508)	Premolar (n = 254)	2nd Molar (n = 127)
Congenital absence	2 (0.4%) ^†††, ‡‡^	14 (5.5%) ***	5 (3.9%) **
Microdonts	1 (0.2%) ^†††^	10 (3.9%) ***^, ‡^	0 (0.0%) ^†^
Short-rooted teeth	52 (10.2%)	23 (9.1%)	9 (7.1%)
Total	55 (10.8%) ^†^	47 (18.5%) *	14 (11.0%)

Chi-square tests with Bonferroni correction were used for comparisons between tooth type in the occurrence of congenital absence, microdonts, and short-rooted teeth. * *p* < 0.05, ** *p* < 0.01 and *** *p* < 0.001 versus incisor or canine or 1st molar; ^†^  *p* < 0.05 and ^†††^  *p* < 0.001 versus premolar; ^‡^  *p* < 0.05 and ^‡‡^  *p* < 0.01 versus 2nd molar.

## Data Availability

The data are available from the corresponding author upon reasonable request.

## References

[1] Iniesta R.R., Paciarotti I., Brougham M.F., McKenzie J.M., Wilson D.C. (2015). Effects of pediatric cancer and its treatment on nutritional status: A systematic review. Nutr. Rev..

[2] GBD 2017 Childhood Cancer Collaborators (2019). The global burden of childhood and adolescent cancer in 2017: An analysis of the Global Burden of Disease Study 2017. Lancet Oncol..

[3] Sakaguchi S., Oda M., Shinkoda Y., Manabe A. (2014). Parents’ perception of pediatric cancer centers in Japan. Pediatr Int..

[4] Kattner P., Strobel H., Khoshnevis N., Grunert M., Bartholomae S., Pruss M., Fitzel R., Halatsch M.E., Schilberg K., Siegelin M.D. (2019). Compare and contrast: Pediatric cancer versus adult malignancies. Cancer Metastasis Rev..

[5] Connelly J.A., Choi S.W., Levine J.E. (2012). Hematopoietic stem cell transplantation for severe congenital neutropenia. Curr. Opin. Hematol..

[6] Li D.Z., Kong P.Y., Sun J.G., Wang X.X., Li G.H., Zhou Y.B., Chen Z.T. (2012). Comparison of total body irradiation before and after chemotherapy in pretreatment for hematopoietic stem cell transplantation. Cancer Biother Radiopharm..

[7] Goho C. (1993). Chemoradiation therapy: Effect on dental development. Pediatr. Dent..

[8] Akitomo T., Kusaka S., Usuda M., Kametani M., Kaneki A., Nishimura T., Ogawa M., Mitsuhata C., Nomura R. (2024). Fusion of a Tooth with a Supernumerary Tooth: A Case Report and Literature Review of 35 cases. Children.

[9] Akitomo T., Asao Y., Iwamoto Y., Kusaka S., Usuda M., Kametani M., Ando T., Sakamoto S., Mitsuhata C., Kajiya M. (2023). A Third Supernumerary Tooth Occurring in the Same Region: A Case Report. Dent. J..

[10] Nishimura S., Inada H., Sawa Y., Ishikawa H. (2013). Risk factors to cause tooth formation anomalies in chemotherapy of paediatric cancers. Eur. J. Cancer Care.

[11] Hernandez M., Pochon C., Chastagner P., Droz D. (2019). Long-term Adverse Effects of Acute Myeloid Leukemia Treatment on Odontogenesis in a Child. Int. J. Clin. Pediatr. Dent..

[12] STROBE Statement. https://www.strobe-statement.org/.

[13] Bilge N.H., Yeşiltepe S., Törenek Ağırman K., Çağlayan F., Bilge O.M. (2018). Investigation of prevalence of dental anomalies by using digital panoramic radiographs. Folia Morphol..

[14] Childhood Cancer WP_SKIP_Project 2022. https://nposuccess.jp/wp-content/uploads/2022/07/Childhood-Cancer-WP_SKIP_Project-2022.pdf.

[15] Erdmann F., Frederiksen L.E., Bonaventure A., Mader L., Hasle H., Robison L.L., Winther J.F. (2021). Childhood cancer: Survival, treatment modalities, late effects and improvements over time. Cancer Epidemiol..

[16] Carrillo C.M., Corrêa F.N., Lopes N.N., Fava M., Odone Filho V. (2014). Dental anomalies in children submitted to antineoplastic therapy. Clinics.

[17] Steliarova-Foucher E., Stiller C., Lacour B., Kaatsch P. (2005). International Classification of Childhood Cancer, third edition. Cancer.

[18] Shirakawa T., Fukumoto S., Iwamoto T., Morikawa K. (2023). Pediatric Dentistry.

[19] Büyükgöze-Dindar M., Tekbaş-Atay M. (2022). Prevalence of Dental Anomalies Assessed Using Panoramic Radiographs in a Sample of the Turkish Population. Chin. J. Dent Res..

[20] Yamasaki Y., Iwasaki T., Hayasaki H., Saitoh I., Tokutomi J., Yawaka Y., Inoue M., Asada Y., Tamura Y., Kanomi R. (2010). Frequency of congenitally missing permanent teeth in Japanese children. Jpn. J. Ped. Dent..

[21] Shum M., Mahoney E., Naysmith K., Macfarlane S., Corbett R., Narsinh M., Natarajan A., Ramadas Y., Hitchings E., Anderson H. (2020). Associations between childhood cancer treatment and tooth agenesis. N. Z. Med. J..

[22] Zarina R.S., Nik-Hussein N.N. (2005). Dental abnormalities of a long-term survivor of a childhood hematological malignancy: Literature review and report of a case. J. Clin. Pediatr. Dent..

[23] Nomura R., Nakano K., Inagaki S., Taniguchi N., Okawa R., Matsumoto M., Ooshima T. (2010). Developmental anomalies of permanent teeth identified in children who received chemotherapy: Report of three cases. Pediatr. Dent. J..

[24] Proc P., Szczepańska J., Skiba A., Zubowska M., Fendler W., Młynarski W. (2016). Dental Anomalies as Late Adverse Effect among Young Children Treated for Cancer. Cancer Res. Treat..

[25] Halperson E., Matalon V., Goldstein G., Saieg Spilberg S., Herzog K., Fux-Noy A., Shmueli A., Ram D., Moskovitz M. (2022). The prevalence of dental developmental anomalies among childhood cancer survivors according to types of anticancer treatment. Sci. Rep..

[26] Jodłowska A., Postek-Stefańska L. (2023). Tooth Abnormalities and Their Age-Dependent Occurrence in Leukemia Survivors. Cancers.

[27] Akitomo T., Kusaka S., Iwamoto Y., Usuda M., Kametani M., Asao Y., Nakano M., Tachikake M., Mitsuhata C., Nomura R. (2023). Five-Year Follow-Up of a Child with Non-Syndromic Oligodontia from before the Primary Dentition Stage: A Case Report. Children.

[28] Kametani M., Akitomo T., Usuda M., Kusaka S., Asao Y., Nakano M., Iwamoto Y., Tachikake M., Ogawa M., Kaneki A. (2024). Evaluation of Periodontal Status and Oral Health Habits with Continual Dental Support for Young Patients with Hemophilia. Appl. Sci..

